# Phenotypic Analysis of Human Lymph Nodes in Subjects With New-Onset Type 1 Diabetes and Healthy Individuals by Flow Cytometry

**DOI:** 10.3389/fimmu.2019.02547

**Published:** 2019-10-31

**Authors:** Jennie H. M. Yang, Leena Khatri, Marius Mickunas, Evangelia Williams, Danijela Tatovic, Mohammad Alhadj Ali, Philippa Young, Penelope Moyle, Vishal Sahni, Ryan Wang, Rejbinder Kaur, Gillian M. Tannahill, Andrew R. Beaton, Danielle M. Gerlag, Caroline O. S. Savage, Antonella Napolitano Rosen, Frank Waldron-Lynch, Colin M. Dayan, Timothy I. M. Tree

**Affiliations:** ^1^Department of Immunobiology, School of Immunology & Microbial Sciences (SIMS), King's College London, London, United Kingdom; ^2^NIHR Biomedical Research Centre, Guy's and St Thomas' NHS Foundation Trust and King's College London, London, United Kingdom; ^3^Diabetes/Autoimmunity Research Group, Cardiff University School of Medicine, Cardiff, United Kingdom; ^4^Public Health Wales, Cardiff, United Kingdom; ^5^Experimental Medicine and Immunotherapeutics (EMIT), Department of Medicine, University of Cambridge, Cambridge, United Kingdom; ^6^GlaxoSmithKline Medicines Research Centre, Stevenage, United Kingdom

**Keywords:** type 1 diabetes, autoimmunity, lymph node, biomarker, immune monitoring

## Abstract

**Background:** Ultrasound guided sampling of human lymph node (LN) combined with advanced flow cytometry allows phenotypic analysis of multiple immune cell subsets. These may provide insights into immune processes and responses to immunotherapies not apparent from analysis of the blood.

**Methods:** Ultrasound guided inguinal LN samples were obtained by both fine needle aspiration (FNA) and core needle biopsy in 10 adults within 8 weeks of diagnosis of type 1 diabetes (T1D) and 12 age-matched healthy controls at two study centers. Peripheral blood mononuclear cells (PBMC) were obtained on the same occasion. Samples were transported same day to the central laboratory and analyzed by multicolour flow cytometry.

**Results:** LN sampling was well-tolerated and yielded sufficient cells for analysis in 95% of cases. We confirmed the segregation of CD69^+^ cells into LN and the predominance of CD8^+^ Temra cells in blood previously reported. In addition, we demonstrated clear enrichment of CD8^+^ naïve, FOXP3^+^ Treg, class-switched B cells, CD56^bright^ NK cells and plasmacytoid dendritic cells (DC) in LNs as well as CD4^+^ T cells of the Th2 phenotype and those expressing Helios and Ki67. Conventional NK cells were virtually absent from LNs as were Th22 and Th1Th17 cells. Paired correlation analysis of blood and LN in the same individuals indicated that for many cell subsets, especially those associated with activation: such as CD25^+^ and proliferating (Ki67^+^) T cells, activated follicular helper T cells and class-switched B cells, levels in the LN compartment could not be predicted by analysis of blood. We also observed an increase in Th1-like Treg and less proliferating (Ki67^+^) CD4^+^ T cells in LN from T1D compared to control LNs, changes which were not reflected in the blood.

**Conclusions:** LN sampling in humans is well-tolerated. We provide the first detailed “roadmap” comparing immune subsets in LN vs. blood emphasizing a role for differentiated effector T cells in the blood and T cell regulation, B cell activation and memory in the LN. For many subsets, frequencies in blood, did not correlate with LN, suggesting that LN sampling would be valuable for monitoring immuno-therapies where these subsets may be impacted.

## Introduction

Type 1 diabetes (T1D) is an autoimmune disease in which a key component of the immune pathology is mediated by T cells (1, 2). Although the disease process is focused on the pancreatic islets, these are not accessible for monitoring in human T1D and therefore studies have focused on changes apparent in the blood.

Despite multiple studies, consistent differences in circulating lymphocyte subpopulations between subjects with and without T1D, including those around disease onset, have been difficult to define. Reports of differences in frequency in CD4^+^, CD8^+^, T follicular helper (Tfh) cells, and subsets of regulatory T cells (Treg) in subjects with new onset T1D (NOT1D) have not always been confirmed although qualitative differences appear to exist (3–8). Extensive efforts have also been directed to monitor islet antigen-specific T cells using techniques including proliferation, cytokine production, ELISPOTs and tetramer stainings with some success (9–17). However, these assays remain challenging due to the low frequency of antigen-specific T cells in the blood (18). The situation is further complicated by the generation of neoepitopes (19, 20).

Lymph nodes (LNs) are important sites in the body where either an immune response to pathogenic antigens or tolerance is initiated (21). In murine models, LNs are routinely used to monitor the immune system as they are easier to access than sampling blood. In the non-obese (NOD) mouse model of T1D islet-specific T cells can be detected in the pancreatic LN before the onset of disease (22) and removal of these LN rescues NOD mice from developing T1D (23). Antigen-specific T cells have also been isolated from pancreatic LNs in humans using organ donor material (24), but this approach cannot be used for serial monitoring. A recent report compared T cell subsets in LNs from organ donors with and without diabetes and reported an increase in Th17 cells and a reduction in Treg (25). However, this did not relate to newly-diagnosed subjects with T1D. Indeed, it has been noted that the pancreatic LN architecture is different in individuals recently diagnosed with T1D, showing a loss of germinal centers and fewer follicular dendritic cell (DC) networks, although the exact significance is unclear (26).

It is possible to serially sample LNs in humans under ultrasound guidance either by fine needle aspiration (FNA) or core biopsy, and this technique is widely practiced in oncology. We recently reported that this approach can detect antigen-specific T cell activation after PPD challenge intradermally (27). Although draining LNs of the target organ could ideally be studied, for systemic autoimmune disease the approach of sampling “distant” LNs (e.g., inguinal) has been used to monitor disease in subjects with rheumatoid arthritis (RA) (28). The authors examined the cellular composition of inguinal lymph nodes (iLNs) in healthy volunteers, by flow cytometry, compared to that from subjects with early RA and individuals at risk of developing RA and showed significant changes in B, CD4^+^, and CD8^+^ T cell composition by flow cytometry analysis between groups. These changes were different from those seen in peripheral blood mononuclear cells (PBMCs) (29, 30), suggesting that studying the composition of immune cells in the iLN has additional value in understanding molecular events in the early phases of an autoimmune disease. Recently, it has been reported in preclinical models of T1D that beta cell derived antigens are released from the pancreas and taken up by other distant LNs (31), suggesting that LNs may also reflect the ongoing disease process.

Monitoring changes in LN populations may be of value for pharmacodynamic analysis of the response to immune therapies, appropriate drug dosing and optimization of risk-benefit in the treatment of autoimmune diseases. Sphingosine-1-phosphate (S1P) agonists (e.g., Fingolimod (FTY720), Ozanimod, Etrasimod, and Amiselimod), as an example, cause sustained downregulation of S1P receptors on lymphoid cells (S1PR1-5) and potentially lymphatic endothelium resulting in lymphopaenia due to sequestration of immune cells in LNs (32). Abatacept (CTLA-4-Ig), which has recently shown success in slowing disease progression in established T1D, binds to CD80/CD86 on antigen presenting cells reducing their ability to deliver co-stimulatory signals and activate T cells. Recent studies have reported biomarkers associated with treatment success including a change in the ratio of CD4^+^ central (Tcm) and effector memory T (Tem) cells (33) and levels of activated B cells (34) which may be due to sequestration of cells in LNs. Furthermore, alemtuzumab (anti-CD52) results in profound depletion of circulating T and B cells, but minimal loss of immune memory responses. Studies in an animal model suggest that this favorable effect is due to failure to effectively deplete immune cells from compartments other than the blood including bone marrow, spleen, and LN (35).

In the current study, we sought to investigate the feasibility and safety of LN sampling as a method to study immune phenotypes in individuals with an organ-specific autoimmune disease—NOT1D—and matched controls. Using multidimensional flow cytometry, we performed a comprehensive analysis of a wide range of leukocytes in paired blood and LN biopsies in order to identify important differences in the frequency, differentiation and activation status of immune cells in the two different compartments that could be relevant for immune interventions. We believe this provides a valuable “roadmap” to indicate when LN sampling is likely to be informative in the development of immunotherapy for autoimmune and other immune-related diseases.

## Materials and Methods

### Study Subjects

Subjects were recruited from two centers, University Hospital Wales, Cardiff and Cambridge University Hospital Trust Foundation (CUHFT), Cambridge, U.K. Ethical approval for this study was granted by Ethics committee and institutional review board, and informed consent was obtained from all subjects enrolled. The study was registered on ClinicalTrials.gov (identifier: NCT02801942). Demographic of subjects screened and enrolled is reported in Table 1. Subjects with T1D had a diagnosis consistent with Type 1a (autoimmune) Diabetes Mellitus (according to ADA and WHO criteria) and were recruited within an interval of up to 8 weeks between the initial diagnosis and the date of biopsy, with residual functioning beta cells as measured by fasted C-peptide levels (≥0.15 nmol/L). Subjects required insulin treatment for at least 7 days prior to the day of biopsy and they were positive at screening for at least one autoantibody associated with T1D (anti-GAD, anti-IA-2, anti-ICA, anti-IAA, and anti-ZnT8) while negative for anti-thyroid peroxidase, anti-tissue transglutaminase and anti-nuclear antibodies. Control subjects had no family history of T1D or any presence of T1D-associated autoantibodies. EDTA blood and iLN FNA and core samples were obtained from 12 control subjects and 10 age-matched individuals with T1D with an average age of 29 and 27, respectively (age range = 19–38). Two follow-up phone calls were carried out ~2–4 days and 7–14 days after the biopsy to enable detection of any post procedural complications and subjects overall experience of the study.

**Table 1:** Details of recruitment efficacy and demographic data of subjects screened and enrolled in the study.

**Number of subjects**	**Control**	**T1D**	**Overall**
Number of subjects planned, [*N*]	10	10	20
Number of subjects screened, [*N*]	23	20	43
Number of subjects biopsied, [*N*]	12	10	22
Number of subjects completed as planned, [*N*] (% of biopsied)	12 (100)	10 (100)	22 (100)
Overall % success rate (%)	52	50	51
**Demographics of enrolled subjects**	**Control**	**T1D**	**Overall**
Age in years [Mean (*SD*)]	28.6 (5.53)	27.0 (5.35)	27.9 (5.38)
**Sex [*****N*** **(%)]**			
Female	4 (33)	4 (40)	8 (36)
Male	8 (67)	6 (60)	14 (64)
BMI (kg/m^2^) [Mean (*SD*)]	24.89 (3.70)	22.68 (2.17)	23.89 (3.23)
Height (cm) [Mean (*SD*)]	174.1 (10.27)	173.4 (10.54)	173.8 (10.15)
Weight (kg) [Mean (*SD*)]	76.18 (17.82)	68.06 (7.60)	72.49 (14.43)
**Ethnicity [*****N*** **(%)]**			
Not hispanic or latino	12 (100)	10 (100)	22 (100)
**Race [*****N*** **(%)]**			
White—White/Caucasian/European Heritage	12 (100)	10 (100)	22 (100)

### Biopsy of iLN FNA and Core

Sampling of iLN was performed under local anesthetic cover and with ultrasound guidance. Both FNA and 16-gauge core biopsy samples were obtained following procedures similar to that described by Tatovic et al. (27) and De hair et al. (36). Using an aseptic technique, between 2 and 5 mL of 1% local anesthetic were injected intra and sub-epidermally, reaching the US-identified LN. The FNA sample was obtained by using a 21-gauge needle. Real-time visualization with ultrasound was used to ensure the needle tip remained within the node. The needle tip was then moved through the nodal cortex to maximize the cell yield of the sample. Following the FNA procedure, a small (2–3 mm) skin incision was made and up to five 16-gauge core biopsies were obtained using the Bard Mission needle or Temno needle, both with manual advance technique to minimize trauma. After obtaining each core sample manual pressure was applied to the area, and at the end of the procedure 5 min of further pressure was applied. This was to ensure hemostasis and reduce the risk of subsequent hematoma formation. Adverse events were reported and are detailed in Table 2.

**Table 2:** Table of adverse events.

**Preferred term**	**Control (*n* = 12)**	**T1D (*n* = 10)**	**Overall (*n* = 22)**
Any event, *n* (%)	9 (75)	5 (50)	14 (64)
Procedural pain	6 (50)	4 (40)	10 (45)
Post procedural contusion	4 (33)	4 (40)	8 (36)
Nausea	1 (8)	0	1 (5)
Fatigue	1 (8)	0	1 (5)

### Sample Processing of iLN FNA and Core

Core iLN samples were homogenized through 70 μm cell strainers using 1 mL syringe plungers. Both core and FNA samples were washed in RPMI and counted using trypan blue. If present, red blood cells were lysed using BD Pharm lysing buffer (BD Pharmingen) and subsequently counted in Türk's solution. In all cases, viability was >95% and FNA and core cell yields are reported in Table 3 [FNA average 0.72 × 10^6^ (range 0.01–3.58 × 10^6^) cells; core average 0.67 × 10^6^ (range <0.01–3.50 × 10^6^)].

**Table 3:** Operator dependent differences in numbers of cells from LN core and fine needle aspirate (FNA) biopsies. Low indicates <0.01 × 10^6^ total cells.

**Site**	**Control/T1D**	**Number of subjects**	**Gender**	**Average age**	**Average number of LN FNA cells (×10^**∧**^6) (range)**	**Number of LN cores**	**Average number of LN core cells (×10^**∧**^6) (range)**
Cardiff	Control	7	4 M 3 F	30	0.96 (0.52–1.60)	4	0.83 (low−3.50)
Cardiff	T1D	5	2 M 3 F	29	1.38 (0.06–3.58)	4	0.59 (0.21–1.21)
Cardiff	Combined	12	6 M 6 F	29	1.13 (0.06–3.58)	4	0.72 (low−3.50)
CUC	Control	5	4 M 1 F	27	0.40 (0.08–1.16)	3	0.16 (0.11–0.25)
CUC	T1D	5	4 M 1 F	25	0.07 (0.01–0.22)	3	0.74 (0.52–1.37)
CUC	Combined	10	8 M 2 F	26	0.23 (0.01–1.16)	3	0.45 (0.11–1.37)
Combined	Control	12	8 M 4 F	29	0.72 (0.08–1.60)	3	0.53 (low−3.50)
Combined	T1D	10	6 M 4 F	27	0.72 (0.01–3.58)	4	0.67 (0.21–1.37)
Combined	Combined	22	14 M 8 F	28	0.72 (0.01–3.58)	3	0.67 (low−3.50)

### Flow Cytometric Analyses

Up to 1 million iLN FNA and core cells (minimum 10,000 cells) were stained in PBS containing 0.2% BSA and 2 mM EDTA using three panels of antibodies, detailed in Supplementary Table 1. Following cell surface staining, cells were fixed and permeabilised using FOXP3/TF staining buffer kit (eBioscience) according to manufacturer's instructions, then stained for FOXP3, Ki67, and Helios. 100–200 μL of EDTA blood was stained using the same three panels of antibodies, however PerFix-nc buffer set (Beckman Coulter) was used for intracellular staining. Blood samples stained only with surface markers were lysed using BD FACS lysing solution (BD Biosciences). Stained samples were acquired on a BD LSRFortessa and data analyzed using FlowJo software (LCC). CS&T beads were run daily and the same machine, with the same cytometer configuration and application settings, was used for the measurements of the samples throughout the study.

### Statistical Analyses

Research analysis plan, study results and primary statistical analysis as per protocol are available at ClinicalTrials.gov (Identifier: NCT02801942). In this manuscript we present a *post-hoc* re-analysis to compare leukocyte frequencies between tissue types and examine frequencies of selected leukocyte subsets with particular relevance to the pathogenesis of T1D. Due to low cell yield obtained from some iLN biopsy samples, the method described by Henley and Keeney (37) was used to exclude results where the number of events acquired was insufficient for accurate enumeration (those with a theoretical CV of ≥20%). Combined iLN data was calculated by taking an average of the frequency data from FNA and core samples, where both data were available. All data were analyzed using R Studio statistical software environment and GraphPad Prism 8 software. Unbiased agglomerative hierarchical clustering analysis was performed with scaled data on all subjects containing complete data for all flow cytometric parameters using complete linkage method and Pheatmap package. Principal component analysis (PCA) was similarly performed using complete scaled data, on a total of 61 populations using base R functions, ggplot2, and Factoextra R packages in an unsupervised approach. When analyzing the full data set to identify populations that differed in frequency between tissues, paired Student's *t*-tests were used to compute *p*-values for all cell populations, correcting for multiple testing using the Holm-Sidak method. When comparing the frequency of individual populations of cells identified as significant in the above analyses separately in T1D and control groups, individual paired tests were used, either Student's *t*-test or Wilcoxon signed-rank test based on the normality of the data. Differences in frequencies between samples from the same tissue (blood or iLN) between individuals with or without T1D were tested using unpaired tests (ANOVA or Kruskal-Wallis test) correcting for testing multiple tissues using Holm-Sidak's or Dunn's multiple comparison test as appropriate. However, due to the low sample size in this study, these tests were not corrected for testing multiple cell subsets. Within individual correlation of cell population frequencies between blood and iLN were calculated by Spearman's rank correlation. Values of *p* < 0.05 were considered statistically significant.

## Results

### LN Biopsy to Investigate Biomarkers of Disease Activity Is Safe, Tolerable and Feasible in Individuals With New Onset T1D

Subject recruitment for this study was carried out at two centers, the Clinical Research Facility at University Hospital Wales, Cardiff (Cardiff), and Clinical Unit Cambridge (CUC). A total of 43 subjects were screened (23 controls and 20 T1D subjects) (Table 1), of which 22 subjects (12 controls and 10 T1D subjects) met inclusion criteria, resulting in screening success rates of 52 and 50%, respectively. All 22 enrolled subjects underwent a biopsy and completed the study as described in the Materials and Methods. The biopsy procedures were very well-tolerated and no serious complications were reported. However, eight contusions spread equally across the control and T1D subjects were reported throughout the study (following LN biopsy) and no hematoma was reported or observed, in contrast to the hematoma rate of 80% reported in De hair et al. (36). Adverse events were reported in 75 and 50% of control and T1D subjects, respectively, all of which were mild in intensity except for a moderate event of nausea reported by one control subject (Table 2). No serious adverse events or any events leading to withdrawal were reported during the study. Overall, combined FNA followed by core biopsy sampling was well-tolerated.

Sampling of both FNA and core biopsies were attempted from all subjects, yielding on average 0.72 × 10^6^ (0.08–1.60 × 10^6^) and 0.72 × 10^6^ (0.01–3.58 × 10^6^) FNA cells and 0.53 × 10^6^ (<0.01 × 10^6^-3.50 × 10^6^) and 0.67 × 10^6^ (0.21–1.37 × 10^6^) core iLN cells from control and T1D subjects, respectively (Table 3), suggesting that overall recovery of cells was similar between biopsy methods and between sites.

### Unbiased Analysis of Immune Cell Subpopulations Suggests Cells Obtained by FNA and Core Biopsy Methods Are Indistinguishable

**Figure 1:**
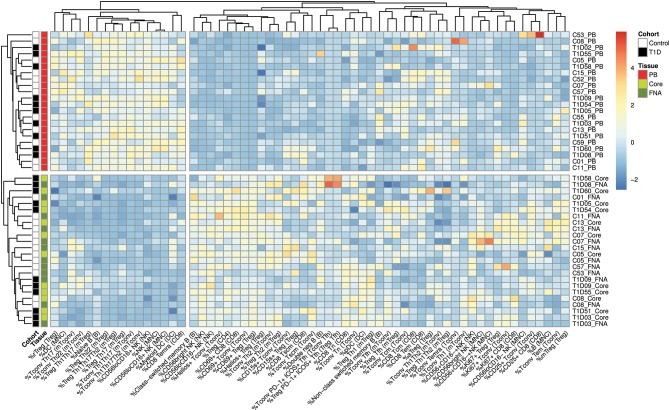
Cells from blood and iLN biopsies display distinct immune profiles. Unbiased Hierarchical clustered heatmap analysis of cell population frequencies (*n* = 61) in peripheral blood (PB), fine needle aspirates (FNA), and core biopsies (Core) from all control and T1D subjects (*n* = 12 and *n* = 10, respectively). The frequency of each population was normalized to the mean frequency from all subjects and tissues and values represent the fold change from mean for each individual sample. Control subjects or those with T1D are indicated with white and black symbols, respectively. Samples from PB, Core and FNA biopsies are depicted by red, light green and dark green symbols, respectively. Individual sample identifiers are shown on the right of the plot indicating cohort group, subject number and biopsy type. Tn, naïve T cells; Tcm, central memory T cells; Tem, effector memory T cells; Temra, Terminally differentiated T cells; Tscm, stem-cell memory-like T cells; rTreg, resting Treg; mTreg, memory Treg; aTreg, activated Treg; Tfh, follicular helper cells; MNC, mononuclear cells; NK, natural killer cells; DC, dendritic cells. Frequencies of cell populations were derived from the cell subset indicated in parentheses.

**Figure 2:**
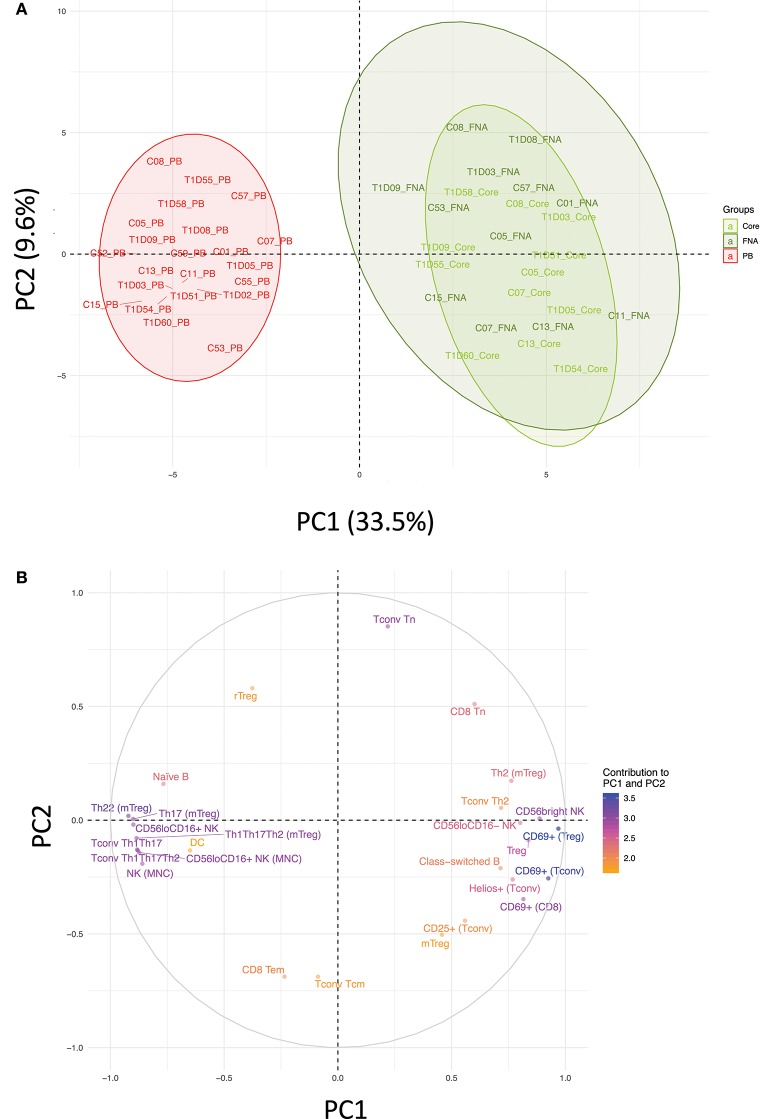
Immune profiles in blood and iLN biopsies differ but cells from FNA and core biopsies are similar. Principle component analysis (PCA) was performed on leukocyte subpopulation frequencies (*n* = 61) in peripheral blood (PB) and iLN biopsies. **(A)** Plot showing the two major principle components of leukocyte subpopulation frequencies from blood, LN fine needle aspirates (FNA) and core biopsies (Core) from all control and T1D subjects (*n* = 12 and *n* = 10, respectively). Individual samples identifiers are as explained in Figure 1. Shaded areas represent 95% confidence ellipses of the mean for each tissue. **(B)** Loading plot indicating how strongly and in which direction each individual variables affect the two major principle components (PC1 and PC2). The top 27 variables contributing to PC1 and PC2 are displayed and their relative influence expressed as a percentage (low% = orange, high% = blue) based on the average contribution of all variables to the PC.

Flow cytometric staining was performed using three panels of monoclonal antibodies designed to measure the frequency of T cell, B cell, DC, monocyte, and NK cell subsets and to assess activation and proliferation of T cells (example gating strategies are shown in Supplementary Figure 1). As monocytes were only observed in blood samples, these were excluded from analysis. In total 61 subpopulations of leukocytes were enumerated from at least one of the two iLN biopsies in all subjects. For some individuals, insufficient cells were recovered from one of the iLN biopsy methods to allow staining with all three antibody panels to be carried out [9 FNA samples and 9 core samples, from which 2 FNA and 1 core samples yielded too few cells to perform any flow staining (7% failure rate)] and we therefore excluded those samples from the following analysis. Unbiased hierarchical clustering analysis of standardized population frequencies was performed to investigate tissue-specific immune phenotypes and a clustered heatmap of these analyses is shown in Figure 1. Blood samples from all 22 individuals (subjects with and without NOT1D) clustered together in a single clade while iLN FNA and core samples were interspersed in another clade, suggesting that immunophenotypes of blood samples are very different to iLN samples and that cells isolated from FNA and core iLN samples had indistinguishable phenotypes. In all cases where sufficient cells were recovered from both iLN biopsies to allow staining with all three panels of antibodies, samples from FNA and core from an individual clustered next to one another, again supporting the similarity of cell populations recovered by these methods. Samples from individuals with T1D and controls were interspersed in each of the two tissue-defined clades and showed no significant disease-associated clustering. These findings were confirmed using principle component analysis (PCA), which revealed a distinct cluster of samples from blood but overlapping clusters from FNA and core samples (Figure 2A). Again, no major disease-associated clustering was observed. The key leukocyte populations driving clustering in the PCA was investigated using a loading plot (Figure 2B). PC1, which describes 33.5% of the variation in the data and may be thought of as the main discriminator between blood and iLN biopsies, was largely driven by differences in T cell subpopulations including those defined by markers of activation, tissue retention and polarization (such as Helios, CD69 and chemokine receptor expression). In contrast, PC2, which describes 9.6% of the variation and may be thought of as the main discriminator between samples from different individuals, was driven mainly by markers of immunological memory and antigen experience. As these unbiased analyses demonstrated that samples from FNA and core were indistinguishable, in all subsequent analyses we used averaged values from FNA and core samples for each subject (iLN).

### Leukocyte Subsets Implicated in the Pathogenesis of T1D Differ in Frequency Between iLN and Blood

**Figure 3:**
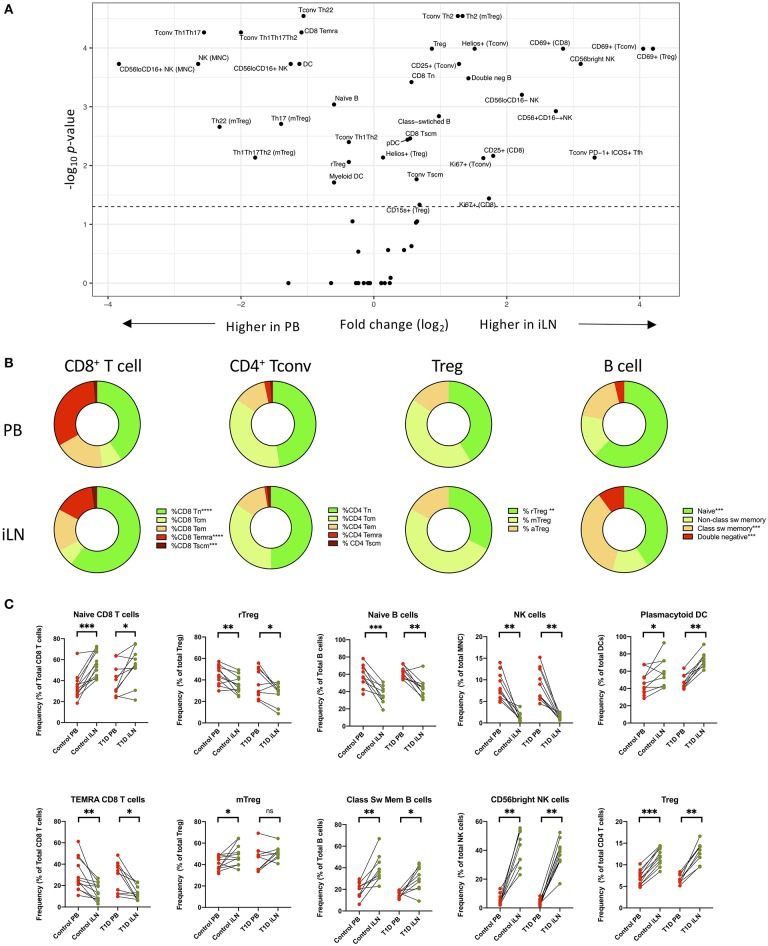
Differences in immune cell subsets between blood and iLN in control and T1D subjects. **(A)** Volcano plot showing fold difference in the frequency of cell populations on the x-axis and significance of the difference on the y-axis (-Log_10_ *p*-values) for blood and combined iLN from pooled control and T1D subjects. *P*-values were calculated adjusting for multiple comparisons using the Holm-Sidak method. **(B)** Doughnut plots showing the distribution of naïve and memory CD8^+^ and CD4^+^ T cells, Treg and B cells in blood and iLN from pooled control and T1D subjects. Significance is calculated as described for panel A. **(C)** Paired frequencies of key individual cell populations showing different frequencies in blood and iLN from control and T1D subjects. Student's *t*-test or Wilcoxon signed-rank test was used. *p < 0.05, **p < 0.01, ***p < 0.001, ****p < 0.0001, ns, not significant; sw, switched.

Next, we performed statistical analysis to identify individual cell populations that differed in frequency between paired samples from iLN and blood using either pooled data from control and T1D subjects or stratifying the data by the two cohorts (correcting for the multiple analyses performed). The results for pooled data are displayed as a volcano plot in Figure 3A (raw data in Supplementary Table 2) which indicates the relationship between the fold difference in frequency of each cell population between blood and iLN and the significance of the change. There are significant changes in the distribution T and B cell naïve/memory populations in all subjects (Figures 3B,C). CD8^+^ T cells demonstrated a higher frequency of naïve and lower frequency of terminally differentiated memory T cells (TEMRA) in iLN compared to blood. In contrast, we observed no difference in the frequency of naïve or memory subsets in CD4^+^ conventional T cells (i.e., non-Tregs; Tconv) but did note a shift in the balance of resting/naïve vs. memory Treg cells, with the former decreased in iLN samples. Higher frequencies of class-switched B cells and lower naïve B cells were also observed in iLN in all subjects. Furthermore, as reported by others (38), we observed a significant reduction in the frequency of NK cells (expressed as a proportion of total mononuclear leukocytes) in iLN compared to blood, but a marked increase in the proportion of NK cells expressing high levels of CD56 (Figure 3C). We also noted a significant increase in the proportion of plasmacytoid DC (pDC) and FOXP3^+^ Treg in iLN. All of these tissue-specific differences in frequency were observed in both T1D and control samples.

### Chemokine Receptor Expression on CD4^+^ T Cells Differs Between iLN and Blood

**Figure 4:**
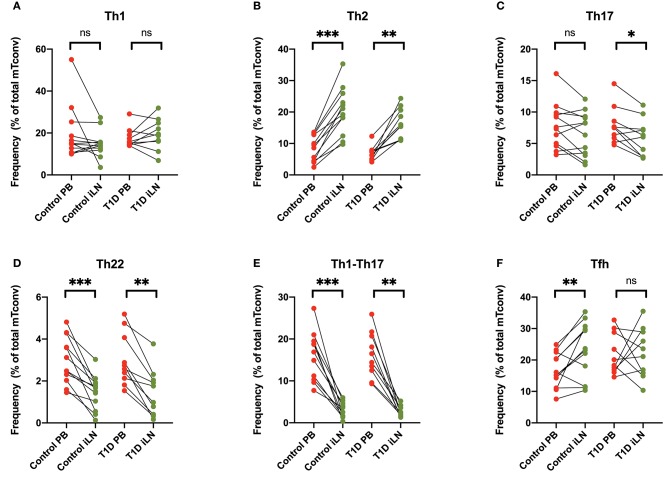
Differences in chemokine receptor expression on memory CD4^+^ T cells between blood and iLN. **(A–F)** Paired frequencies of CD4^+^ helper T cell populations in blood and iLN from control and T1D subjects. Frequencies of each cell type are expressed as a percentage of total memory CD4^+^ conventional T cells (i.e., FOXP3^−^ CD4^+^ T cells). Student's *t*-test or Wilcoxon signed-rank test was used. *p < 0.05, **p < 0.01, ***p < 0.001, ns, not significant.

Differential expression of chemokine receptors can be used to identify CD4^+^ T cells with different helper function potential (39). We therefore categorized CD4^+^ Tconv cells based on patterns of chemokine expression (Figure 4) using the gating scheme shown in Supplementary Figure 1. In all subjects, we observed a significant increase in cells characterized by expression of CCR4 in the absence of CXCR3, CCR6, CCR10, or CXCR5 (Th2-like) in iLN compared to blood (Figure 4B). By contrast, we observed a significant decrease in iLN in subpopulations of cells characterized by expression of CCR6 including those co-expressing CCR10 (Th22) or CXCR3 (Th1-Th17) in both cohorts (Figures 4C–E). Cells expressing CXCR5 were identified as Tfh and although they were detected at higher frequency in iLN compared to blood when considering control individuals, they were not significantly higher in iLN from individuals with T1D or when the total cohort was analyzed (Figure 4F and Supplementary Table 2).

### Markers of Cellular Activation and Proliferation Are Increased in T Cells From iLN

**Figure 5:**
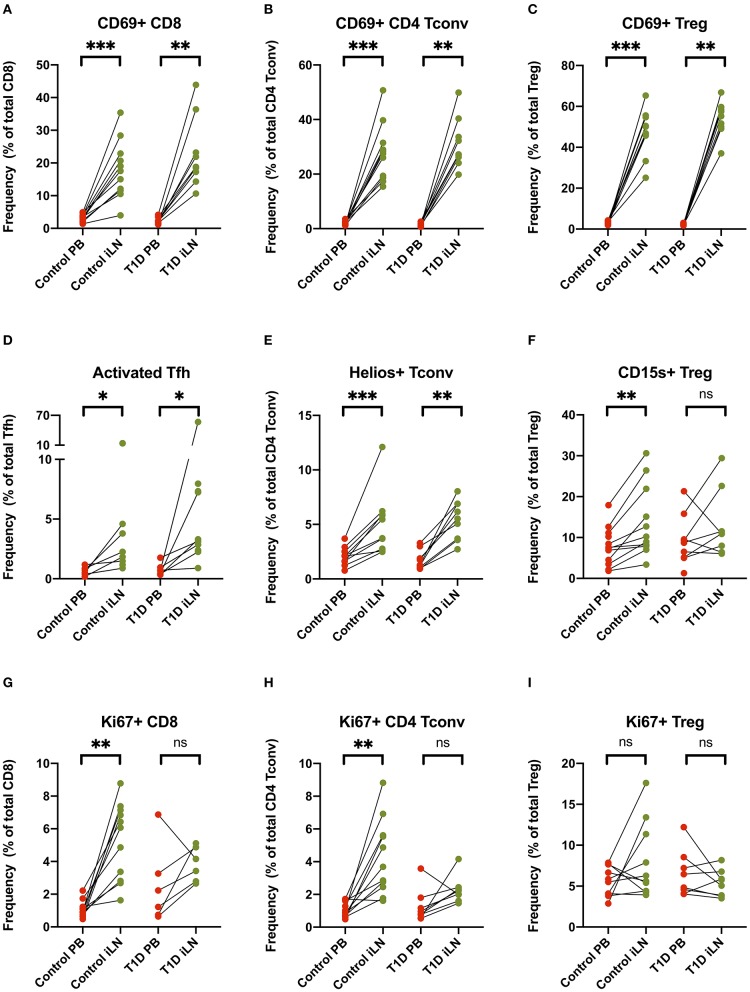
T cell subsets in iLN show enhanced expression of markers associated with activation, proliferation and tissue retention compared to blood. **(A–I)** Paired frequencies of CD4^+^ helper T cell populations in blood and iLN from control and T1D subjects. Frequencies of each cell type are expressed as a percentage of total T cell subtype. Student's *t*-test or Wilcoxon signed-rank test was used. *p < 0.05, **p < 0.01, ***p < 0.001, ns, not significant.

Others have reported increased expression of the early activation/tissue retention marker CD69 on CD8^+^ and CD4^+^ T cells from LNs compared to blood (27, 29, 30), a finding we also observed and extended to FOXP3^+^ Tregs in both study cohorts (Figures 5A–C). Similarly increased CD25 expression is observed in both CD8^+^ and CD4^+^ Tconv cells in LNs compared to blood (Supplementary Figure 2). In addition, in all subjects, we observed an increase of markers of cellular activation in iLN CD4^+^ T cells including ICOS and PD-1 co-expression in Tfh and increased expression of the Helios transcription factor in Tconv (Figures 5D,E). Interestingly, when we examined expression of CD15s (Sialyl Lewis x), which identifies highly differentiated and the most suppressive Tregs (40), we observed a higher level of expression in iLN Tregs from control individuals but did not observe this in subjects with T1D (Figure 5F). Consistent with the LN being a major site for priming of T cells, we also observed a significant increase in the proportion of CD8^+^ and CD4^+^ Tconv cells expressing the marker of cellular proliferation, Ki67, although this only reached significance for the samples from control individuals and not for those with T1D (Figures 5G,H). In contrast, no increase in Ki67 expression was observed in Tregs from either study cohort or when all subjects were analyzed together suggesting that, in contrast to non-Treg, proliferation of FOXP3^+^ Tregs is not enriched in LNs compared to blood (Figure 5I).

### Some Cell Populations Show Strong Correlation Between Frequency in Blood and iLN, Others Show No Correlation

**Figure 6:**
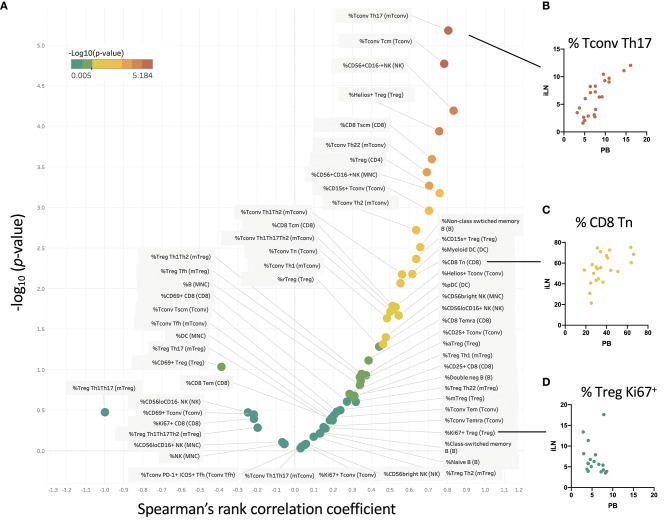
Some cell populations show strong correlation between frequency in blood and iLN, others show no correlation. **(A)** Within individual correlation of cell population frequencies between blood and iLN was calculated using data from all individuals by Spearman's rank correlation. Points are colored based on *p*-values with those showing significant correlation (*p* < 0.05) shown in yellow-red symbols and those with no significant correlation shown in shades of green. **(B–D)** Example correlation for individual cell subsets showing a high **(B)**, medium **(C)**, and no significant correlation **(D)** between frequencies in the blood and iLN.

We examined within individual correlation of the frequency of each cell subset between blood and iLN. This was to determine whether, although differing in absolute frequency, assessing the frequency of a particular cell population in blood would be a good surrogate for the level in LN. This analysis revealed that whilst for some populations there was good correlation between the frequency observed in blood and iLN (e.g., CD4^+^ Tconv central memory or Helios^+^ Treg), for other cell populations there was little or no correlation between the frequency in blood and iLN (e.g., naïve B cells or memory Treg) (Figure 6 and Supplementary Table 3). Correlation was consistently poor in all proliferating (Ki67^+^) T cells as well as other populations characterized by recent activation, such as aTfh or aTreg.

### Subtle Alterations in Treg Subsets Are Observed in iLN but Not Blood in T1D

**Figure 7:**
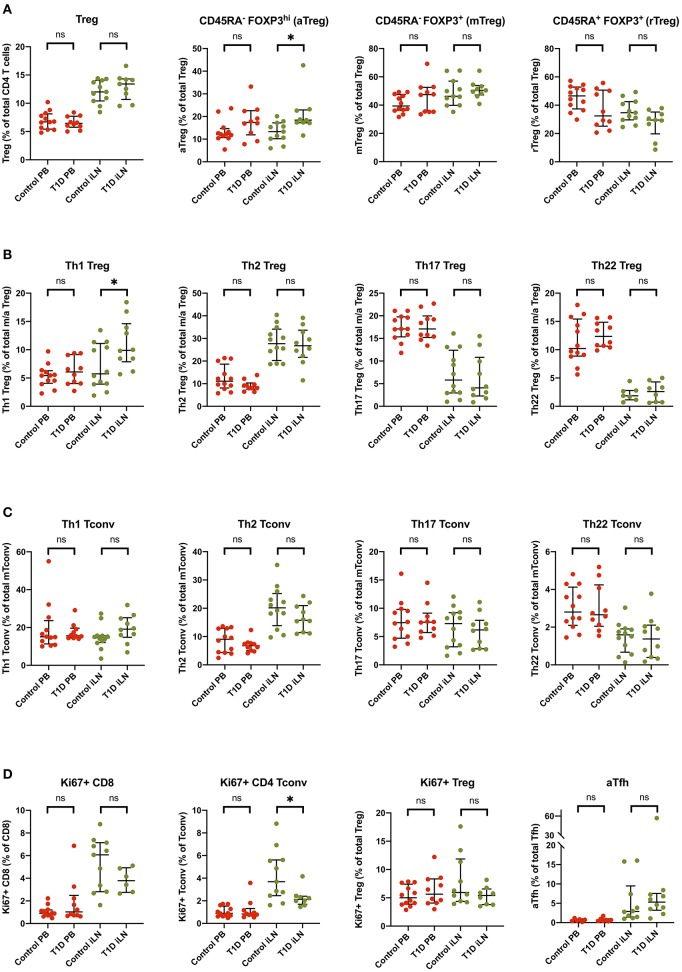
Subtle alterations in the balance of T cell subpopulations between T1D and control individuals are evident in iLN but not in blood. Individual frequencies of **(A)** naïve/memory Treg cell subsets **(B)**, T helper-like Treg subsets **(C)**, conventional helper T cell subsets, and **(D)** proliferation and activation marker on T cell subsets in blood and iLN from control and T1D subjects. Frequencies of each cell type are expressed as a percentage of total T cell subtype. Error bars represent median and inter quartile ranges for each population. ANOVA or Kruskal-Wallis test was used. *p<0.05. ns, not significant.

Several reports have noted alterations in the frequency of Treg subsets in blood from individuals with new onset T1D including markers of memory, activation and polarization (6, 41), however, these differences are generally modest in size and hence require large group sizes to detect them. Consistent with the majority of studies, we did not observe a difference in the total frequency of FOXP3^+^ Tregs in individuals with T1D in either blood or iLN (Figure 7). We did, however, observe subtle differences in the balance of Treg subsets in the iLN samples between control and T1D subjects. Individuals with T1D had higher frequencies of activated, antigen-experienced Tregs (defined as CD45RA^−^ FOXP3^hi^) (Figure 7A) and also a higher proportion of Th1-like Tregs compared to these from control subjects (Figure 7B). No such difference was observed between the two cohorts in helper T subsets in either blood or iLN (Figure 7C). Finally, we observed a lower proportion of CD4^+^ Tconv cells expressing Ki67, a marker of cell proliferation, in iLN of individuals with T1D compared to control subjects (Figure 7D). All of these differences were only observed in iLN but not in blood samples.

## Discussion

The recirculation of lymphocytes was first described by Gowans in 1959 (42) and emphasizes the dynamic interchange between tissues, secondary LN organs, and blood. However, the convenience of blood for sampling in humans has led immunologists to largely ignore changes in the other immune compartments in studying immune responses and immune-mediated diseases. Here we add to the limited literature on human LN sampling to demonstrate that ultrasound guided LN sampling is well-tolerated and can be reliably performed on LNs that are not pathologically enlarged with a >95% success rate when performed by experience radiologists. Both FNA and core biopsy techniques are relatively low risk procedures, although the former carries less risk of bleeding and is potentially more suitable for repetitive sampling. However, whereas the FNA provides only cytological information, core biopsy can provide additional histological information. Furthermore, studies have suggested that FNA biopsy may not accurately reflect the composition of the whole LN due to sampling of selected regions (43). Since excisional biopsy of iLN was not appropriate in this context, we compared FNA with core biopsy, with an average of 3 cores/individual, hypothesizing that core biopsy may be more representative of the whole LN. However, we observed no significant differences in the phenotype of cells obtained from paired samples suggesting either technique yields similar results. Comparison of blood and LN from the same individual shows striking differences in the leukocyte populations present, suggesting any blood contamination during the procedure is minimal.

Advances in simultaneous multicolour flow cytometry in recent years have allowed us to map differences in leukocyte subpopulations between LN and blood in greater detail and with greater resolution than was previously possible (27–30). We have clearly shown that in the CD8^+^ T cell compartment, naïve cells predominate in the LN while highly activated Temra cells predominate in the blood confirming previous observations (27). In the CD4^+^ T cell compartment, the frequency of naïve and memory cells is similar in LN and blood, Temra cells are rare in both compartments, but stem cell-like memory cells are more common in LNs (Supplementary Table 2). Interestingly, when expressed as a proportion of all CD4^+^ T cells, Tregs are twice as frequent in LN than in blood and there is an increase in the proportion of memory and decrease in resting (or naïve) Tregs in LN compared to blood. By contrast, naïve B cells are more frequent in the blood than in the LN while class-switched memory B cells are more common in LNs. This emphasizes a role for more differentiated effector T cells in the blood while LNs appear to play a key role in CD4^+^ T cell proliferation, T cell regulation, B cell activation and memory. Treg function was compared by *in vitro* co-culture suppression assays as previously described (44) using matched iLN and blood samples. No difference in the suppressive capacity of Tregs between tissues or between patients and controls were observed (data not shown); however, due to the limited number of cells isolated from non-pathologically enlarged iLN biopsies, we were only able to perform assays on four subjects with T1D and 4 controls, limiting statistical power.

Other leukocyte subsets in addition to lymphocytes showed polarization between blood and LN. The most striking example is NK cells, in which conventional CD56^dim^ NK cells are virtually absent from LN while CD56^bright^ NK cells are greatly enriched. CD56^bright^ NK cells are the major producer of NK cell cytokines and constitutively express the high affinity heterotrimeric IL-2 receptor making them responsive to picomolar levels of this cytokine *in vitro* and *in vivo* (45). IL-2 treatment is currently being tested to treat a variety of inflammatory and autoimmune diseases by targeting Tregs (46), however, several studies have shown that even using very low doses IL-2 treatment also activates and expands the frequency CD56^bright^ NK cells in blood (44, 47) but to date no data is available on whether a similar expansion is also seen in the LNs. The balance of DC subpopulations is also altered between blood and iLN, with pDC enriched in the latter. These cells are major producers of type 1 interferons in response to viral or other TLR ligands and play a key role in activating CTL and Th1 responses. There is growing evidence that numerical expansion or excessive activation of pDCs may play a role in the pathogenesis of a number of autoimmune conditions (48, 49) including T1D (50, 51), however these studies have not investigated pDC phenotype or function in LNs, the site where these cells are likely to directly influence downstream adaptive immune responses.

The availability of additional T cell markers, especially chemokine receptors, has allowed us to explore the functional differences between the blood and LN compartments in more detail than previously. In particular we observed that while Th1-like cells are similarly distributed between blood and LN, Th2 cells are enriched in the LN (consistent with B cell activation). A trend toward more Tfh cells is also observed in the LN. In contrast, cell populations expressing CCR6 (especially Th1Th17 and Th22 cells) are enriched in the blood. CCR6-expressing cells are thought to play a major role in a number of Th17-mediated autoimmune diseases including T1D, psoriasis and RA and targeting these cells or the cytokines they produce is an area of active clinical trial activity (52, 53). In addition to confirming the very high degree of segregation of CD69-expressing cells into LNs (27, 30), we see an increase in other activation markers in LN T cells, notably CD25, Helios and Ki67, even bearing in mind that the LNs sampled were not selected to be actively involved in an acute immune response.

Via comparative analysis of the correlation of blood and LN levels across all different leukocyte subsets studied, we have for the first time demonstrated that although blood sampling is likely to provide a good indication of LN levels of certain cell populations, for others it does not. Of particular relevance to immune monitoring in settings of autoimmune and inflammatory diseases or immunotherapy, we noted correlation between blood and LN was consistently poor for all populations of proliferating T cells and also other cell populations associated with recent activation, such as ICOS^+^PD1^+^ Tfh (aTfh) and CD45RA^−^FOXP3^hi^ Treg (aTreg). We also noted poor correlation for both CD4^+^ and CD8^+^ Tem cells as well as all B cell subsets.

Although subject numbers were small, limiting statistical power, our study allowed us to compare T cell subsets in LNs between subjects with active organ-specific autoimmunity (NOT1D) and healthy age-matched controls. Consistent with the relatively low levels of autoimmune T cell activation (54) and the highly antigen-specific nature of T1D, any differences in lymphocytes subsets were more modest than seen in previous reports in RA (28, 29), where an increase in CD8^+^ memory and antigen-experienced T cells in lymphoid tissue was seen along with an increased frequency of non-circulating or recently activated (CD69^+^) CD8^+^CD45RA^+^ T cells and an increased frequency of (CD69^+^) CD8^+^CD45RO^+^ T cells in blood. In our comparisons, no significant differences were seen for any of the subsets in the blood between subjects with T1D and controls. However, we did observe an increase in Th1-like Treg and a reduction in proliferating (Ki67^+^) CD4^+^ and CD8^+^ T cells in T1D LNs compared to control LNs. Interestingly, a similar increase in Th1-like Tregs was recently described by Viisanen et al. in the blood of children with NOT1D (55), however the group sizes needed to reveal this difference (*p* = 0.01 with *n* = 73 T1D and *n* = 166 control) were much higher than is used in this study supporting the hypothesis that, when present, disease-associated immune phenotypes may be more easily detected in LN than in the blood. A trend toward an increase in activated Tfh cells in LN was observed, which is possibly unexpected in view of the disruption of germinal centers previously reported in pancreatic LNs in T1D (26). Note that the significance of these changes has not been corrected for testing multiple populations and hence should be viewed with caution.

Our results do provide the first “roadmap” of when LN sampling would be most informative as predicted in Figure 6. They suggest, for example, that blood monitoring of Treg therapies (e.g., low dose IL-2), aimed at inducing Treg proliferation, or depletion/repopulation therapies (such as anti-thymocyte globulin or alemtuzumab), which aim to reset the balance of memory-naive T cell subsets, may fail to represent what is happening in the LNs. The same may be true for the effects of drugs that target CCR6 expressing (Th17-family) cells, such as ustekinumab (which particularly targets Th1-Th17 cells), or anti-CD20 therapies which target B cells or anti-CD3 which targets T cells. Although our phenotyping was extensive, we did not obtain enough material to look for additional phenotypic indicators such as cytokine expression. Ramwadhdoebe et al. reported reduced IL-17 and interferon gamma positivity in CD8^+^ cells from LN in RA but observed no changes in the blood. Finally, we found it challenging, given the small size of the LN in NOT1D and healthy controls to obtain informative histology from the core biopsies. Future studies may allow single cell transcriptomic analysis of LN-derived cells to provide additional information.

In summary, ultrasound guided LN sampling is well-tolerated and has a high success rate in experienced hands. Although differences in autoimmunity at the cell population level are modest, it is likely to provide valuable information for immunotherapy via cell manipulation and should be considered in mechanistic analyses and targeted proof of pharmacology of novel interventions.

## Data Availability Statement

The raw data supporting the conclusions of this manuscript will be made available by the authors, without undue reservation, to any qualified researcher. Anonymized individual participant data and study documents can be requested for further research from www.clinicalstudydatarequest.com.

## Ethics Statement

The studies involving human participants were reviewed and approved by London—Dulwich Research Ethics Committee. The patients/participants provided their written informed consent to participate in this study.

## Author Contributions

JY, LK, MM, and EW performed experiments and analyzed data. JY and LK performed statistical analysis. JY, TT, and CD wrote sections of the manuscript. DT, FW-L, CD, and TT contributed to the design of the study. MA contributed to patient recruitment and follow ups. PY and PM provided clinical samples. TT is the guarantor of this work, and as such, had full access to all of the data in the study and takes responsibility for the integrity of the data and the accuracy of the data analysis. All authors contributed to manuscript revision, read and approved the submitted version.

### Conflict of Interest

VS, RW, RK, GT, AB, DG, CS, and AN are or were employees and shareholders of GlaxoSmithKline. The funder was involved in the study design, collection, analysis, interpretation of data, the writing of this article and the decision to submit it for publication. TT has received research support from GSK and has served on an advisory board for GSK. CD has served on an advisory board for GSK. The remaining authors declare that the research was conducted in the absence of any commercial or financial relationships that could be construed as a potential conflict of interest.
