# Supplementary material for: Phenotypic Analysis of Human Lymph Nodes in Subjects With New-Onset Type 1 Diabetes and Healthy Individuals by Flow Cytometry
Source: Front Immunol. 2019 Oct 31;10:2547. doi: 10.3389/fimmu.2019.02547 (PMC6842967; doi:10.3389/fimmu.2019.02547)
Supplement: Supplementary file 1 [file Table_1.docx]

Supplementary Material

Phenotypic analysis of human lymph nodes in subjects with new-onset type 1 diabetes and healthy individuals by flow cytometry

**Jennie H.M. Yang^1,2*^, Leena Khatri^1,2^, Marius Mickunas^1,2^, Evangelia Williams^1,2^, Danijela Tatovic^3^, Mohammad Alhadj Ali^3^, Philippa Young^4^, Penelope Moyle^5^, Vishal Sahni^6^, Ryan Wang^6^, Rejbinder Kaur^6^, Gillian M. Tannahill^6^, Andrew R. Beaton^6^, Danielle M. Gerlag^6^, Caroline O.S. Savage^6^, Antonella Napolitano Rosen^6^, Frank Waldron-Lynch^5^, Colin M. Dayan^3^ and Timothy I.M. Tree^1,2*^**

**Supplementary table 1. Details of antibodies used for multiparameter flow cytometry.**

**Supplementary table 2.** Observed mean fold difference in the frequency of cell populations with standard error (S.E.) and significance of the difference for blood and combined iLN from pooled control and T1D subjects. *P*-values were calculated adjusting for multiple comparisons using the Holm-Sidak method. Populations found at a higher frequency in iLN are shown in green and those at a higher frequency in blood in red.

**Supplementary table 3.** Within individual correlation of cell population frequencies between blood and iLN were calculated using data from all individuals by Spearman’s rank correlation. Table shows significance and rank correlation coefficient (R^2^). Cells are coloured based on significance with those showing strongest correlation shown in red and those with lowest correlation in green.

**Supplementary figure 1. Gating strategies for flow cytometric panels.** (A-B) Representative staining profile for (A) blood and (B) iLN using antibodies from the Treg panel. (C-D) Representative staining profile for (C) blood and (D) iLN using antibodies from the T cell panel. Non-Tn cell were considered as total memory cells, which were used to derive frequencies of different helper T (Th Tconv) subsets and Th-like Treg subsets. (E-F) Representative staining profile for (E) blood and (F) iLN using antibodies from the APC panel. DN = Double negative; sw = switched; mem = memory. Placement of flow gates for CD4^+^ conventional Tcm and Tem cells in iLN was challenging due to low CCR7 staining resolution as compared to that for blood samples. Review of the primary analysis of study results (available at ClinicalTrials.gov (Identifier: NCT02801942)) unexpectedly showed lower frequency of Tconv Tcm cells in iLN compared to blood. Hence, these gates were re-adjusted, and then further reviewed and reached consensus by two analysts.

1. Peripheral Blood

**Supplementary figure 1. Gating strategies for flow cytometric panels.**

1. iLN

**Supplementary figure 1. Gating strategies for flow cytometric panels.**

1. Peripheral blood

1. iLN

**Supplementary figure 1. Gating strategies for flow cytometric panels.**

1. Peripheral blood

1. iLN

**Supplementary figure 2. CD8^+^ and CD4^+^ Tconv cells in iLN show increased expression of CD25 compared to blood.** (A) Paired frequencies of CD25^+^ CD8^+^ T cell in blood and iLN from control and T1D subjects. (B) Paired frequencies of CD25^+^ CD4^+^ Tconv cell in blood and iLN from control and T1D subjects. Frequencies of each cell type are expressed as a percentage of total T cell subtype. Student’s *t*-test was used. ** *p*<0.01, *** *p*<0.001, **** *p*<0.0001.

(A) (B)
